# 

**Published:** 1845-08

**Authors:** 


### CONTENTS

### OF NO. II.

---

### ORIGINAL COMMUNICATIONS.

### ESSAYS AND CASES.

Art I.—Some account of Epidemic Erysipelas as it prevailed in some parts of Ohio. By John Dawson, M.D., of Jamestown, Ohio. - - - - - 93

### SELECTIONS FROM AMERICAN AND FOREIGN JOURNALS

Homœopathy in Italy, - - - - - - - - 155

Preservative Influence of Vaccination, - - - - - 159

Practical Remarks on the Use of Carbonate of Lead, in the form of White Paint, in the treatment of Burns and Scalds. By Samuel D. Gross, M.D., Professor of Surgery in the Louisville Medical Institute. - - 160

Practical Observations on the various forms of Dyspepsia. By Robert Dick, M.D. - - - - - - - 164

On the Effects of Blood as an Antidote for Arsenic. - - 171

Treatment of Chronic Mammary Abscess by the Breast Pump and Syringe. - - - - - - - - - 173

Poisoning by Cherry Kernels. - - - - - - - 173

Professor Corneliani on the Proximate Cause and Treatment of Chlorosis. - - - - - - - - - - 174

Gun-Shot Wounds of the Hip—the Ball passing around the Back. - - - - - - - - - - 175

ORIGINAL INTELLIGENCE.

Letter from the Senior Editor. - - - - - - 176
Astounding Scientific Discovery. - - - - - - 182
Health of the City. - - - - - - - - - 184
Louisville Summer School of Medicine. - - - - 184
Books Received. - - - - - - - - - 184

